# Reply to Masutani et al—Protective Factors That Maintain Asymptomatic Longevity in Untreated Congenitally Corrected Transposition of Great Arteries

**DOI:** 10.1016/j.cjco.2021.12.011

**Published:** 2021-12-30

**Authors:** Kohei Osakada, Masanobu Ohya, Kenji Waki, Hiroshi Nasu, Kazushige Kadota

**Figure 1:**
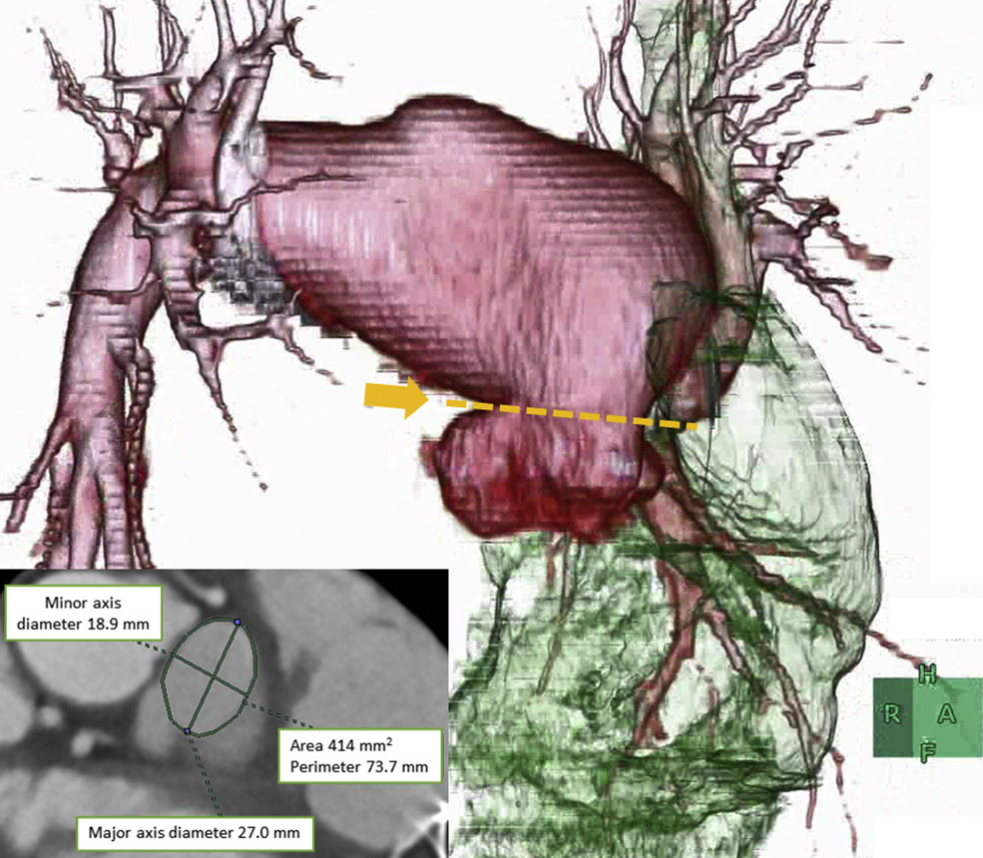
A 3-dimensional computed tomography image showing the pulmonary artery in a systolic phase from a right anterior oblique angle of 30 degrees, which revealed a significant constriction (**yellow arrow**) and subsequent dilation of the main pulmonary artery. The measured values of the constricted part were as follows: area, 414 mm^2^; perimeter, 73.7 mm; major axis diameter, 27.0 mm; and minor axis diameter, 18.9 mm.

To the Editor:

We appreciate the insightful comments from Professor Masutani and his colleagues. We agree that the presence/absence of pulmonary stenosis (PS) is important in this case. We performed a reanalysis using computed tomography (CT) images. Figure 1 is a 3-dimensional CT image showing significant constriction and subsequent dilation of the main pulmonary artery. Although additional reexaminations, including echocardiography, should have been performed, we were unable to take this course of action because the patient, unfortunately, had died by the time of writing. As a result, we could not determine the presence or absence of PS.

Helsen et al. reported that in patients with congenitally corrected transposition of the great arteries, those with PS with a pressure gradient of ≥ 30 mm Hg had a better prognosis than those without PS.^1^ One of the main reasons that PS has been thought to improve prognosis is that it prevents the development of systemic atrioventricular valvular regurgitation by inducing deviation of the interventricular septum toward systemic ventricular.^2^^,^^3^ In this case, the measured values of CT images showed that the constricted portion of the main pulmonary artery was not narrow, and an echocardiogram showed that the mitral regurgitation pressure gradient was 36 mm Hg. The degree of PS, if any, was therefore expected to be relatively mild.
